# Changing Habits With the Happy Hands App: Qualitative Focus Group Study of a Hand Osteoarthritis Self-Management Intervention

**DOI:** 10.2196/82773

**Published:** 2026-02-02

**Authors:** Kristine Aasness Fjeldstad, Anne Therese Tveter, Eivor Rasmussen, Lena Olden, Sissel Nyheim, Thalita Blanck, Rikke Munk Killingmo, Ingvild Kjeken

**Affiliations:** 1 Health Service Research and Innovation Unit Center for Treatment of Rheumatic and Musculoskeletal Diseases (REMEDY) Diakonhjemmet Hospital Oslo Norway; 2 Faculty of Health Sciences Department of Rehabilitaion Science and Health Technology OsloMet – Oslo Metropolitan University Oslo Norway; 3 Hand Therapy Clinic Kirkenes Hospital Kirkenes Norway; 4 Department of Physiotherapy and Occupational Therapy Levanger Hospital Levanger Norway; 5 Norwegian Rheumatism Association Oslo Norway

**Keywords:** mHealth, eHealth, osteoarthritis, hand exercises, self-management, social cognitive theory, qualitative research

## Abstract

**Background:**

People with hand osteoarthritis represent a large patient group with limited access to recommended treatment. In recent years, there has been a notable shift in health care delivery, with increased use of digital technologies. The Happy Hands app (The University Information Technology Center [USIT]) is a digital self-management intervention developed to provide evidence-based treatment for people with hand osteoarthritis, with the goal of empowering them to self-manage their disease. Participants’ experiences and perceptions of using this digital intervention are crucial for the adoption and continued use of the Happy Hands app.

**Objective:**

The objective of this qualitative study was to explore participants’ experience with using the Happy Hands app, focusing on whether and how it empowered them to self-manage their hand osteoarthritis.

**Methods:**

The study is embedded within a randomized controlled trial (RCT). The participants were recruited from the intervention group in the RCT, who got access to the Happy Hands app. The 12-week self-management intervention included a hand exercise program and informational videos about hand osteoarthritis. Focus groups were conducted in various geographical areas in Norway. The focus groups were transcribed verbatim, coded, and analyzed inductively using reflexive thematic analysis.

**Results:**

Seven focus groups, with a total of 26 participants, were recruited from both specialist and primary health care. The mean age was 67 years. Three themes were developed from the analysis. The first theme, “Being acknowledged,” highlights the essential role of recognition for people with hand osteoarthritis. It suggests that the Happy Hands app provided participants with a sense of validation and support. The second theme, “Changed perception of hand osteoarthritis,” indicates that participants gained insights and knowledge about their condition. This new understanding empowered them to make more informed decisions about their care, fostering a sense of hope and motivation by demonstrating that effective measures are available to manage the disease. The third theme, “Changing habits with the Happy Hands app,” describes how participants developed new habits after using the self-management intervention delivered through the app. The exercise program was experienced as motivating, flexible, well-structured, and committing. Some challenges were reported, including experiencing pain during or after exercising. The new habits included performing hand exercises and implementing ergonomic working methods, which were tailored to meet the individual needs and integrated into the participants’ daily lives and routines.

**Conclusions:**

The findings suggest that the Happy Hands app is a valuable tool for supporting people with hand osteoarthritis in managing their disease by helping them integrate hand osteoarthritis management into their daily lives.

**Trial Registration:**

ClinicalTrials.gov NCT05568875; https://clinicaltrials.gov/study/NCT05568875

## Introduction

Hand osteoarthritis is a prevalent and debilitating condition. Nearly half of all women and a quarter of all men will develop hand osteoarthritis during their lifetime [1], with symptoms often emerging around middle adulthood. People with hand osteoarthritis typically experience pain, stiffness, and reduced grip strength [2]. These symptoms challenge individuals’ ability to perform everyday activities, such as household tasks, hobbies, and work, and may reduce health-related quality of life. Although there is no cure for hand osteoarthritis, various effective measures can help alleviate symptoms [3]. Access to treatment is, however, often limited, and the quality of care is suboptimal [4], with patients often being referred to surgery without having received recommended treatment [5].

First-line treatments for people with hand osteoarthritis include patient education, hand exercises, and the use of assistive devices [3,6]. Patient education is central, as much of the management of hand osteoarthritis relies on actions individuals have to perform themselves. However, supporting individuals in self-managing a chronic condition like hand osteoarthritis can be challenging [4], particularly in maintaining adherence [7].

There has been a significant shift in the delivery of health care in recent years, with an increased emphasis on the use of digital devices in the provision of care. Government documents have highlighted eHealth as a key strategy to optimize resource use, streamline patient pathways, and ensure that individuals get access to health information [8].

Previous studies have demonstrated that digital delivery for hand osteoarthritis is a good alternative to traditional in-person consultations [9] and effective in improving hand function and reducing pain compared to usual care [10]. Qualitative studies have described participants being mostly positive to digital delivery of osteoarthritis management [11-13].

The use of mobile apps offers a valuable tool for ensuring access to treatment and supporting individuals in self-managing their hand osteoarthritis [6]. Mobile apps can offer tailored information and guided exercise routines, which may also improve exercise adherence [14,15].

The Happy Hands app (The University Information Technology Center [USIT]) contains a 12-week self-management intervention for people with hand osteoarthritis, developed to ensure access to evidence-based treatment. The goal of the app is to empower people with hand osteoarthritis to self-manage their disease [15]. Before this study, the app underwent feasibility testing aimed at refining and improving its design. The results indicated that the Happy Hands app contributed to reductions in pain and stiffness, as well as improvements in activity performance and grip strength. In focus group interviews, participants reported that the app was useful and highlighted several areas for further enhancement [15]. In this study, the aim was to explore participants’ experience with using the Happy Hands app, focusing on whether and how it empowered them to self-manage their hand osteoarthritis.

## Methods

### Study Design

This qualitative study is conducted as part of a randomized controlled trial (RCT) investigating the effect and cost-effectiveness of the Happy Hands app. Focus groups were conducted with participants from the intervention group in the RCT to explore their experiences of using a digital intervention for hand osteoarthritis.

### Happy Hands App

The Happy Hands app was developed by a research group at Diakonhjemmet hospital [15]. The development of the app was guided by social cognitive theory [16]. The behavior change taxonomy, a classification system categorizing different techniques used to change behavior [17], was used to classify the elements in the app.

The app contains a self-management intervention with informational videos and a hand exercise program. The informational videos include different themes, such as information about hand osteoarthritis, hand exercises, use of assistive devices and orthoses, medication and surgical options, and how to cope with everyday life. Furthermore, the self-management program includes a hand exercise program with videos showing how the patients should warm up and perform exercises to improve mobility, strength, and coordination, and a stretching exercise. The informational videos and exercise program are delivered in a progressive order across 12 weeks. The participants could choose 3 days a week to use the app. On these designated days they received notifications on their smartphones with that week’s informational videos and hand exercises. The participants had the opportunity to tick off each completed exercise. Encouragement, motivational messages, and quizzes were provided each week to enhance continued adherence to the intervention [18].

### Setting

This qualitative study was nested within the Happy Hands study, a multicenter RCT that evaluated whether a self-management intervention delivered through the Happy Hands app, in addition to usual care, was more effective than usual care alone for people with hand osteoarthritis [18]. The RCT was conducted between November 2022 and February 2024. Participants in the RCT were recruited from 14 hospitals, 2 rehabilitation centers, 3 physiotherapy clinics, and 1 private rheumatology center, following their regular consultations at these sites. In total, 376 participants were enrolled and randomly allocated to either the control group (n=185) or the intervention group (n=191).

The control group received a one-page information sheet and usual care. Usual care for people with hand osteoarthritis varies across settings and can include no treatment, a one-day educational course, or consultations with an occupational therapist and/or a rheumatologist. The intervention group received the same one-page information sheet and usual care; in addition, they were given access to the Happy Hands app, which provided them with a 12-week self-management program.

### Participants and Recruitment

In the RCT, eligible participants had symptomatic hand osteoarthritis diagnosed by health care personnel, owned a smartphone, and could read and understand Norwegian. Participants were excluded from the RCT if they had cognitive deficits, were scheduled for hand surgery within 3 months after inclusion, or had serious comorbidities or inflammatory rheumatic disease. Participants were recruited through clinical practice. Clinicians applied the inclusion and exclusion criteria to determine eligibility. Information on serious comorbidities was obtained through participant self-report, and cognitive function was assessed through clinical evaluation.

Participants in the focus groups were recruited from the intervention group in the RCT. When they were enrolled in the RCT and signed the consent form, participants could indicate their willingness to be contacted for an interview about their experiences with the app. A purposive sampling strategy was used to ensure diversity in geographic location and health care setting. Participants were recruited from various regions across Norway to ensure geographic diversity and from both primary and specialist care. Individuals in the intervention group who consented to an interview were contacted by phone. In total, 28 participants agreed to participate. However, 2 of the participants declined before the focus groups were conducted due to unknown reasons. Thus, a total of 26 participants participated in 7 focus groups.

The focus groups took place between 7 and 11 months after recruitment for the RCT began. All focus groups were conducted in 2023, the first 6 in June and the last one in October. We chose this timing to ensure that participants had completed the intervention, enabling us to explore their reflections on the full intervention period. One participant, however, joined a focus group after one month because of late enrollment, whereas the rest were interviewed after 3 or more months. Adherence to app use throughout the 12-week program period was not an inclusion criterion.

### Data Collection

Focus groups were chosen as the data collection method in this study because they are useful to obtain in-depth understanding of a topic by taking advantage of the group dynamics and discussions that can occur in a group setting [19].

All 7 focus groups were conducted by the first author (KAAF). Either the last author (IK), a master student associated with the project, or one of the clinicians working at the recruitment sites assisted as moderators in the focus groups by taking notes and asking follow-up questions. KAAF is a female PhD candidate and nurse with some experience in qualitative research, while IK is an occupational therapist with extensive experience in both clinical practice and research. The master student is also an occupational therapist and was writing a thesis concerning the Happy Hands app. The clinicians were working as either a physiotherapist or occupational therapist at the recruitment sites and assisted in recruiting patients in their clinical practice to the RCT and have experience working with individuals with hand osteoarthritis. KAAF had previously met a few of the participants in connection with their inclusion in the RCT, while IK and the master student had no prior relationship with the participants before the study. The clinicians had previously met some of the participants during consultations.

The focus groups were held at the site where the participants were recruited to the RCT, at a hospital or clinic, except for one focus group that was held in a conference room at a hotel. Before the focus groups began, participants were informed about the aim of the study. They were told that any experience, both positive and negative, they had had with the Happy Hands app was welcomed and were encouraged to discuss and ask each other questions. This aligns with Liamputtong [20], who describes that focus groups allow for more topics to be initiated by participants, as they steer the conversation more than the researcher does in individual interviews.

A first version of the interview guide was developed by KAAF, ATT, and IK and focused on participants’ experiences, problems with using the app, perceived benefits, and future use (Multimedia Appendix 1). It was reviewed by patient research partners (SN and TB), who suggested adding a question about initial experiences, including whether users received advice and support. They also recommended using the term “challenging” instead of “difficult” to frame questions more positively. The interview guide was thereafter tested in a pilot focus group. As no major revisions were necessary, the data from this session was included in the final dataset.

At the end of the focus group, the participants were asked if they had anything to add. Each focus group consisted of 3-5 participants and lasted between 50 minutes and one and a half hours. The audio recordings were transcribed verbatim by KAAF and a research assistant. Notes were taken by the moderator during each focus group. Following each session, the moderator and interviewer (KAAF) discussed their impressions of the content and clarified any points that required further understanding.

We applied the concept of information power to determine whether we had a sufficient sample size. This approach suggests that factors such as the study aim, participant specificity, theoretical framework, interview quality, and analysis strategy should guide decisions about when sufficient information has been obtained [21]. Our study sought to explore user experience in a broad context, which typically necessitates a diverse sample. We therefore decided to include focus groups from different regions in Norway, covering various levels of care. Accordingly, 6 focus groups were conducted in different geographical areas, encompassing both primary and specialist health care settings. We anticipated that this strategy would yield sufficient data to address our research questions. Following the initial round of analysis, we noted a lack of information regarding negative experiences with the app and how it would be used in the future. To strengthen our understanding of this aspect, we conducted one additional focus group. We then considered that the focus groups provided a comprehensive understanding of the participants’ experiences and perspectives. The data had sufficient depth and richness to address the study aim, and thus, we concluded that information power was achieved*.*

### Theoretical Framework

Following an initial phase of inductive analysis and coding, social cognitive theory (SCT) [16] was applied to explore whether participants had benefited from the intervention and achieved behavior change. SCT outlines key determinants for behavior change: knowledge about health risks, outcome expectations, goal-setting and strategies to achieve them, and perceived facilitators and barriers. A crucial aspect is self-efficacy, which is the belief that individuals have in their ability to achieve change through their actions [16]. Additionally, observational learning, learning a new behavior by watching others perform it, is a central concept [22].

To complement this behavioral perspective, the common-sense model of illness representations developed by Leventhal et al [23], was also applied to the analysis in this study. While SCT focuses on the mechanisms underlying behavior change, the Common-Sense Model helps to explain how individuals understand and make sense of their illness. It posits that illness representations are shaped by various sources of information, such as societal lay knowledge, advice from health care professionals, and personal experiences with the illness. Illness representation can be divided into 5 different dimensions. “Cause” considers beliefs about what is causing the disease. “Consequences” addresses beliefs regarding its impact, “identity” is beliefs regarding the symptoms, and “timeline” refers to beliefs about the duration and progress. “Cure or control” is the belief about the potential for recovery or management and the individual’s ability to influence their condition. These dimensions collectively shape how patients perceive their illness and have significance for the way individuals will seek help and for adopting a coping strategy for the disease [24].

### Involvement of Patient Research Partners

Two patient research partners (SN and TB), both of whom completed the 12-week app program, were involved from the outset of the study and contributed to the review of the interview guide. Following a presentation of the results, a discussion was held between them and IK and KAAF. The patient research partners indicated that they identified with the results and the developed themes. They are both coauthors of this article and have reviewed and provided feedback on the manuscript.

### Data Analysis

Reflexive thematic analysis was applied to analyze the interviews, aiming to explore, interpret, and develop patterns of meaning across the dataset. This method involves 6 key phases, including familiarizing oneself with the data, generating initial codes, constructing themes, reviewing themes, defining and naming themes, and producing the final report. A key concept in this method is reflexivity, which involves continuously reflecting on our assumptions and practices and how these affect the research [25]. Analysis was performed by the first author (KAAF), the second author (ATT), and the last author (IK). Throughout the research process, we actively engaged in reflexivity through several discussions. These discussions enabled us to examine our perceptions and how they might have influenced the research.

After conducting the first 6 focus groups, we performed an initial analysis by reading through the transcripts multiple times. The aim of this familiarization phase was to gain an overall understanding of the data. Notes were written throughout this process to identify interesting elements and possible patterns across the dataset and as a tool to be able to reflect on interpretations [25]. Following this familiarization phase, we also decided to conduct one additional focus group.

Each transcript was coded by KAAF using NVivo software (QSR International Pty Ltd). Text segments relevant to the aim of the study were tagged with distinct codes for different meanings. The initial analysis was conducted inductively. Coding was performed at a semantic level, staying close to the participants’ language**.** In a second round of coding, a deductive approach was applied, looking for segments of text relevant to the theories we planned to include. The second and last authors (ATT and IK) each read half of the transcripts, and the team met to discuss reflections and interpretations on content in data and potential themes.

The codes were gathered, and 3 initial themes were developed from them. The tentatively developed themes were reviewed against all the codes clustered around each theme. A discussion was held with the patient research partners, IK and KAAF, following a presentation about the results. They expressed recognition of the developed themes and highlighted the importance of feeling seen and heard, access to information, and how exercises had become a natural part of their daily routines, facilitated by the app. Quotes were selected from the transcripts to illustrate the findings. Finally, the themes were defined and given names. All coauthors reviewed the drafts and provided feedback.

Reflexive notes were written throughout the research process to increase awareness of preconceptions and to enhance transparency. Initial preconceptions were documented at the beginning of the study and later compared with the final results. The considerable differences between them can be understood as an indication that the first author’s preconceptions did not prevent new insights from emerging.

### Ethical Considerations

This study was approved by the Regional Committees for Health Research Ethics (477746), the Data Protection Officer (00660), and the local research committee at Diakonhjemmet hospital. Informed consent was obtained from all participants. The interviews were audio recorded by the Nettskjema-Diktafon app (University of Oslo), which safely transfers encrypted audio files to Services for Sensitive data (TSD) at the University of Oslo. TSD is a platform for collecting and storing sensitive data in compliance with the Norwegian privacy regulation. The qualitative study is briefly described in the trial description for the RCT registered on ClinicalTrials.gov (NCT05568875). We followed the Consolidated Criteria for Reporting Qualitative Research (COREQ; Multimedia Appendix 2) [26]. Participants did not receive compensation for taking part in the focus groups but were offered reimbursement for travel expenses.

## Results

### Participants’ Characteristics

A total of 26 participants (18 women and 8 men) participated in 7 focus groups. The mean age was 67 years. Participants had hand osteoarthritis diagnosis for between one and 25 years (Table 1).

**Table 1:** Participant characteristics (N=26).

Characteristic			Participants
Age (years), mean (SD)			67 (7)
**Gender, n (%)**			
	Women	18 (69)	
	Men	8 (31)	
**Years living with hand osteoarthritis, n (%)**			
	0-9	13 (50)	
	10-19	11 (42)	
	More than 19	2 (8)	
**Geographical area, n (%)**			
	Southeast	15 (58)	
	West	4 (15)	
	Central	3 (12)	
	North	4 (15)	

Through the analysis we developed 3 themes. The theme “Being acknowledged” highlights the importance informants placed on having their hand osteoarthritis recognized and validated. Meanwhile, the theme “Changed perception of hand osteoarthritis” reflects how their perceptions of hand osteoarthritis changed after gaining insights from the self-management intervention in the app. Finally, “Changing habits with the Happy Hands” app reports how people with hand osteoarthritis integrated hand exercising and ergonomic working methods into their daily life and routines.

### Being Acknowledged

A discussion about a feeling of not getting support and recognition for their hand osteoarthritis emerged in one of the focus groups. A few participants described a feeling of not receiving adequate health care. For example, one participant described having had pain for several years and experiencing a prolonged wait before finally receiving a diagnosis:

I’ve actually had pain for many years, but no one understood what it was. [...] I go to the doctor, no, it's not that, I take tests, nothing happens, but this year I got the diagnosis.

Woman, 57 years

In one focus group it was discussed that osteoarthritis is a disease that is given little attention in society, even though it affects many people. The participants described lacking support from the workplace and that they did not talk much about their disease with family or friends. Moreover, participants described a sense of dealing with the burden of hand osteoarthritis alone:

It is not talked about a lot, you go around with pains and you don’t talk about it. We’re supposed to manage it, it’s not a problem, we women can fix it.

Woman, 72 years

Being part of the project and getting access to the app was therefore perceived as meaningful, as these experiences provided a sense of validation and acknowledgment. This was described in one of the focus groups.

*I want to take a slightly different approach, which has actually been my problem. I’ve had pain and limitations in activity for a very long time, and that leads to, now I’m almost about to cry, poor sleep, and a low mood for a long time […] you feel like it’s just something we have to live with […]So when [the doctor] said that I could be part of this, I was really happy […] That someone cares and someone sees you. Yes.* [Woman, 75 years]

Furthermore, the focus group revealed that after getting the app and becoming part of the Happy Hands study, participants were increasingly willing to discuss hand osteoarthritis with their friends and family, having previously avoided doing so.

But what is currently the most important thing is all my female friends […] it has started to become a topic of conversation. […] There have been times when we’ve sat together and done some exercises at gatherings, saying, ‘Now we’re stretching our fingers,’ and turning it into a little show.

Woman, 72 years

This willingness to share experiences with others reflects an increased openness about the condition, which may contribute to a sense of acknowledgment and support.

The theme highlights how some participants perceive a lack of support for their hand osteoarthritis, making their involvement in the study and access to the app feel like a genuine acknowledgment of their condition. This experience encouraged them to open up about their struggles with friends and family, indicating that it developed a sense of community and support.

### Changed Perception of Hand Osteoarthritis

Through the self-management intervention delivered through the app, participants gained knowledge and insights about hand osteoarthritis, which seemed to have changed their perception of the disease in different ways. Participants described that the app gave them hope and motivation by demonstrating that different measures could be taken to manage the disease effectively.

It’s important that one can do something about it, because I think it is what has been most discouraging in a way, that there isn’t anything to do about it. It just has to run its course. And then, just the fact that you see that there is something you can do.

Woman, 65 years

Moreover, knowledge about the disease and its mechanisms reduced the feeling of uncertainty, for example, by getting an understanding of why they had pain and why some activities suddenly were difficult to perform.

You are now more aware of why it is there, right [...] what is it now, what have I done, so the uncertainty is gone, now you sort of know what you have to deal with.

Man, 66 years

The participants emphasized that it was important to receive knowledge about recommended treatment options for patients with hand osteoarthritis. This could be information about surgery, medication, use of orthoses and how to modify activity performance. This information could empower them to make informed decisions about their care based on updated knowledge. For example, in one of the focus groups, it was a consensus that they appreciated knowing that surgery is recommended as the last treatment option, since they did not want to have surgery. One participant was referred to surgery but decided to wait and see if he would benefit from doing hand exercises.

So, there were a number of things that became clear now […] it was like, you were given the steps and that surgery was presented as the last option, right. […] And... I liked that, because I don't want to be cut open.

Man, 66 years

A few participants described gaining knowledge that using their hands can have a preventive effect and that it is not harmful to use their hands even when experiencing pain:

I was a bit skeptical at first because I was afraid that doing something painful might be harmful. I thought I was causing damage, so I was very cautious. But now, I am willing to tolerate some pain as long as it doesn’t get worse. In a way, it’s reassuring to know this, and it has helped me normalize the experience.

Woman, 64 years

This theme describes how participants gained valuable insights about hand osteoarthritis, which shifted their perceptions of the disease. They expressed that increased knowledge about treatment options and their understanding of their condition alleviated uncertainty, supported them to make more informed decisions about their care, and provided hope and motivation by demonstrating that there exist measures that can help manage the disease.

### Changing Habits With the Happy Hands App

This theme explores how participants changed their habits after completing the self-management intervention in the Happy Hands app, particularly in exercising and activity performance.

#### Developing New Habits: Exercising

The app’s exercise component helped participants learn hand exercises and integrate them into their daily lives. Participants unanimously agreed that it was easy to use the app, with no one reporting any difficulties in understanding its technical aspects. Many participants emphasized the flexibility of the app as a key factor in its success, appreciating the ability to perform the exercises anywhere and at a time that was convenient for them.

You can exercise almost anywhere. I have exercised outdoors, I have exercised indoors in different places, I’ve kind of brought along a small box with equipment. It is very practical not to be tied to a specific table or chair or anything like that.

Woman, 74 years

The participants described that the app instilled a sense of commitment by requiring them to complete specific exercises within a designated timeframe. This sense of obligation was further reinforced by their participation in a research project and the requirement to tick off each completed exercise within the app. Several participants expressed that receiving notifications on their smartphones served as helpful reminders to complete their exercises. Moreover, scheduling 3 fixed days per week provided structure and consistency.

The app includes videos that demonstrate how to perform the exercises, which participants noted improved their ability to execute them correctly. A few even described feeling a sense of connection with the person in the videos, as if they were exercising alongside someone, which they found motivating.

I think it's very important that you see a person demonstrating the exercises, rather than just sitting and reading something. For me, there’s a significant difference between the two. […] The physical or psychological—I'm not sure exactly what to call it—connection makes a big difference when it comes to actually doing those exercises.

Woman, 72 years

The possibility to track their progress in the app, complete quizzes, and receive feedback was also described as a motivation by some of the participants. Moreover, these features encouraged them to put in extra effort to both understand and retain the app’ content.

Participants in the focus groups reported experiencing various improvements. When they experienced improvement, for instance, in improved strength, mobility, or ability to perform activities, they were motivated to carry out the training program.

I found it very motivating when I noticed that, wow, I have much more mobility, it didn’t come the first week, not the second week, and not the third week, but maybe in the fourth and fifth week I started to get better. I can bend all my fingers now, which I couldn’t do before, and then you understand, this is working, and it is also motivating to continue.

Woman, 57 years

There were, however, also some challenges described when it came to using the Happy Hands app. These included experiencing pain during or after exercising. Some participants said that their hand osteoarthritis was too advanced for effective exercising. Not everyone reported improvement. It was suggested that the app should include guidance on how to manage pain during exercise.

But my experience has been, in short, that I have a few fingers that have very mild osteoarthritis. And I can feel how good this app is for those fingers. At the same time, I notice that it’s not very good for my bad fingers, as they actually get provoked and irritated by some of them [the exercises].

Woman, 65 years

There were also other reasons for not continuing to perform hand exercises. For example, one participant said she had stopped doing exercises simply because she had more going on in her life and she had forgotten about it. Other diseases or injuries could also take away the focus of conducting hand exercises. Additionally, some participants expressed that they wished that new exercises had appeared in the app, as this would have been motivating.

The results suggest that the Happy Hands app enabled participants to learn an exercise program for hand osteoarthritis, to integrate it into their daily routines, and ultimately to establish it as a habit. Once the participants had memorized the exercises, it was no longer necessary to watch the app while performing them. This made it possible to integrate exercising into their daily life by performing them while engaged in other activities.

Now I notice that I sit and watch the news while doing it with a tennis ball and perform many of the exercises a bit automatically in my daily life. When you go for a walk, […] many of them are easy to continue with.

Woman, 67 years

They could also adjust the exercising to when and how it suited the individual. For example, some preferred following the exercise program less structured. Other participants preferred to continue to exercise in a structured way, 3 times a week with the app in front of them.

Moreover, the participants adjusted the exercises because of other factors. For example, conducting exercises with less intensity when experiencing pain or conducting exercises differently when finding the exercise difficult to carry out.

… squeezing that ball has also started to hurt more, as I said you should squeeze as hard as you can for five seconds, five times, per hand. So, I'm trying to be a bit more careful; I’m still trying to do it and hold it, but maybe I’m not squeezing as hard simply because it hurts more than it did before.

Man, 59 years

The majority of the participants said they planned to continue to exercise. These results suggest that the app facilitated making exercising a habit.

This theme discusses how the app helped participants integrate hand exercises into their daily routines. Participants found the app easy to use and appreciated its flexibility. A sense of commitment was supported by the need to complete the exercise on time, reminders from the app, and scheduled sessions. Seeing a person demonstrate the exercises gave participants motivation, as well as making them easier to understand. Many experienced improvements that enhanced motivation to continue. However, some participants faced challenges, such as pain during or after exercising or osteoarthritis too advanced to benefit from exercise. The app facilitated the development of new habits for many participants, enabling them to integrate exercises into their daily life and adjust them to their individual needs and circumstances.

#### Developing New Habits: Adapting Daily Activities

The findings also reveal that participants made changes to how they performed everyday activities, including ergonomic working methods and use of assistive devices as suggested in the app. The adaptations they learned through the app made some activities easier to perform. For example, participants reported learning new techniques for holding and lifting objects, such as avoiding carrying items solely with their fingers or warming up their hands before use. These changes seemed to have had a significant impact on their daily life, as one participant described,

I do notice a difference, yes, [...] and then I think about warming up when I'm going to write, for example. Once I’ve warmed up, it’s not so embarrassing to write my name anymore. It was embarrassing [...] it was almost like being naked, I would say, sixty-seven, and I could hardly write my name.

Man, 67 years

Additionally, participants found the information about different assistive devices to be helpful. For example, they mentioned tools like a nutcracker for opening soda bottles, as well as other devices such as a cheese cutter and a bread knife with ergonomic handles. However, a few participants expressed some reluctance to using assistive devices, as they associated them with “being old.”

This theme described how participants also changed their performance of everyday activities after insights they had gained from the app.

## Discussion

### Principal Findings

The aim of this study was to explore participants’ experience with using the Happy Hands app, focusing on whether and how it empowered them to self-manage their hand osteoarthritis. Our findings indicate that participants previously had experienced a lack of support and recognition for their hand osteoarthritis. Hence, to become part of the study and get access to the app provided them with a sense of validation and acknowledgment. Furthermore, the results suggest that participants’ illness perception changed after gaining insights from the informational videos in the app, from viewing their condition as something insignificant and beyond their control to recognizing its importance, taking their own experiences seriously, and gaining insights into strategies they could use to alleviate pain and improve function.

Participants perceived the app as flexible, motivating, and well-structured, which facilitated both their learning and adherence to the exercise program as well as alternative working methods. As a result, they developed new habits, such as integrating hand exercises into their daily life and changing the way they perform everyday activities. However, for some participants, their condition hindered their ability to complete the exercises, often due to pain.

The findings in our study reveal that some participants felt unsupported and lacked recognition in their struggles with hand osteoarthritis by family members, health care providers, and society at large. This lack of understanding and acknowledgment is consistent with findings from other qualitative studies. Hill et al [27] noted that individuals with hand osteoarthritis perceive a lack of empathy from health professionals regarding the impact of the disease, whereas Gignac et al [28] found that symptoms were often dismissed as a normal part of aging. In our study, participants reported minimizing the severity of their condition, rarely discussing their hand osteoarthritis despite its significant impact on their daily life. This tendency to downplay symptoms aligns with the findings of Magnussen et al [29], who reported that people with hand osteoarthritis often feel undeserving of health care. Bukhave and Huniche [30] also found that participants did not seek medical care, despite experiencing a wide range of activity limitations. According to the common-sense model of illness representation [23], people’s perceptions and understanding of their disease influence their behaviors and coping strategies. It is therefore essential for health care professionals to explore patients’ previous experiences with the health care system and to actively acknowledge their perceived symptoms and functional challenges. Feeling that their condition is validated may have shifted participants’ views from considering their hand osteoarthritis as insignificant and something they simply had to endure to recognizing its importance and the impact it has on their daily lives.

As outlined in the common-sense model of illness representations, various kinds of knowledge contribute to a person’s perception of their illness [24]. The findings in our study suggest that participants changed their illness perception by acquiring new knowledge. They gained expert knowledge through the app, where health professionals provided valuable information about hand osteoarthritis. Additionally, they developed new personal knowledge through their own experiences, such as noticing improvement and regaining the ability to perform activities. This suggests that the newly acquired knowledge enabled participants to develop a new understanding of their illness, resulting in a shift in their illness perception.

According to the common-sense model of illness representations, illness perception can be categorized into different dimensions, including the “cause” dimension, which refers to what people believe to be the underlying reason for their illness [24]. Our findings suggest that participants altered their perception of this dimension by using the app, transitioning from uncertainty and fear about the origins of their pain, worried that activity might worsen their condition, to a more informed understanding. They came to recognize the actual cause of their illness and understood that using their hands would not exacerbate their pain.

Our results indicate that participants’ perceptions shifted from believing nothing could be done about the disease to recognizing that effective measures are available. This aligns with the “cure or control” dimension of the common-sense model of illness representations, which describes a person’s belief about how a disease can be managed [24]. The initial perception that nothing can be done aligns with findings from other studies, which report that individuals often view hand osteoarthritis as a natural result of wear and tear or aging [27,31], and believe that the condition is untreatable [29]. Participants in our study also changed their perception of their own ability to take action. For instance, information about treatment options helped them understand that delaying surgery is often a recommended approach. This shift in perception, from viewing hand osteoarthritis as untreatable to acknowledging the availability of effective management strategies, empowered participants to begin adopting these strategies.

An important finding in our study is that participants developed new habits by integrating hand exercises into their daily routines and adjusting them to their individual needs. The results further suggest that the Happy Hands app played a key role in facilitating this behavioral change. SCT, which is widely used in the development of health interventions [22], may help explain how the Happy Hands app contributed to this change. By supporting participants to gradually learn and implement the exercise program, the app enabled them to master activities that were previously difficult.

The findings further reveal that due to new behaviors, such as hand exercising, participants experienced positive outcomes, including reduced pain and an improved ability to perform previously challenging activities. In SCT, self-efficacy, a person’s belief in their ability to make necessary changes to achieve a desired outcome, is a central concept. Self-efficacy can be enhanced by empowering individuals to succeed with achievable actions that progressively become challenging [22]. Thus, the Happy Hands app may have played a significant role in enhancing participants’ self-efficacy, which is critical for driving sustained behavior change [16].

According to SCT, self-regulation is a key strategy for facilitating successful behavior change. It comprises several elements, including self-monitoring, goal setting, feedback, and self-reward, and often includes the possibility to observe and record one’s behavior [22]. These elements were integrated into the app and were highlighted by participants as important in helping them adhere to the exercise program. Participants emphasized the value of tracking their progress by ticking off completed exercises, which fostered a sense of commitment. Moreover, having 3 designated exercise days per week provided structure, while app notifications served as helpful reminders to complete the exercises. This aligns with the goal-setting element of SCT, which suggests that setting short-term, achievable goals motivates effort and guides actions [16]. Furthermore, participants found motivation in the app’s feedback features, such as being able to track their progress and answer quizzes. Feedback is recognized in SCT as a crucial component in facilitating behavior change, reinforcing effort, and sustaining motivation.

The app includes videos that demonstrate how to perform the exercises, which was appreciated by the participants. First, observing someone performing the exercises helped them understand how they should be performed correctly. Second, participants expressed that doing the exercises while watching the videos felt as though they were training alongside someone, which increased their motivation. This finding can be further explained through SCT, where observational learning is a key concept, suggesting that seeing others perform a specific behavior encouraged app users to replicate that behavior [22].

Most participants expressed a positive attitude toward the intervention delivered through the Happy Hands app. However, some challenges were reported. These included experiencing pain during or after exercising, and for some participants, their hand osteoarthritis seemed too severe for them to benefit from the exercises. Such individuals may require individual face-to-face guidance, either as a substitute for or in combination with digitally delivered interventions. While this raises concerns about the usability of digital interventions for all patients, it is important to recognize that widespread use of digital solutions could free up valuable health care resources for those who require more intensive follow-up care from health professionals.

People with hand osteoarthritis are a large patient group with limited treatment options. To ensure a sustainable health care service, the integration of digital solutions and technologies is essential. The Happy Hands app has the potential to provide people with hand osteoarthritis access to reliable information, guidance, and recommended treatment.

### Strengths and Limitations

A key strength of this study is that participants were recruited from both primary and specialist care and from various regions across Norway, ensuring representation across a broad geographical area and levels of care. Furthermore, we collaborated with patient research partners with hand osteoarthritis who had used the Happy Hands app for 12 weeks. These partners provided feedback on the interview guide and participated in a meeting to discuss the study results. They expressed that they could personally relate to the findings. Respondent validation, that is, sharing results with people who have experienced the phenomena being studied, serves as an important strategy to help prevent misinterpretations [32].

The focus groups consisted of participants who shared their perceptions and experiences, with the interviews being transcribed verbatim. This approach facilitated the collection of rich data, characterized by depth and diversity, thus providing a comprehensive understanding of the issues being studied [32]. Another strength was the collaborative analysis process, where both the PhD candidate and supervisors read through transcripts, participated in coding, and engaged in discussions regarding the content in the interviews. Such discussions and feedback are beneficial for identifying potential flaws in logic or methodology, thereby enhancing the rigor of the research [32].

Our study also has some limitations. One is that participants voluntarily chose to participate in the Happy Hands study and agreed to be interviewed. This may have introduced a selection bias, as they might have had a more positive attitude toward using an app and were more receptive of digitally delivered interventions. This could also indicate that the participants in our study had a higher level of eHealth literacy compared to the general population. Individuals who did not use the app may have been more likely to decline participation in the focus group interviews. Future studies should aim to include their perspectives and experiences, as this could yield valuable insights into whether and how the app could be improved to meet their needs, as well as inform alternative approaches to care delivery.

While focus groups are useful for exploring collective experiences and generating rich, interactive data, they also have limitations. Group dynamics can influence what is shared, with dominant voices potentially influencing the discussion. Some individuals may hesitate to express dissenting or personal views, particularly when discussing sensitive topics. Additionally, the time constraints of group discussions may limit the depth of individual narratives [20].

### Conclusions

The findings indicate that the Happy Hands app initiated and facilitated a process that resulted in behavior change among the participants. The app provided validation and acknowledgment of the disease. Furthermore, insights from the app contributed to a more informed understanding of hand osteoarthritis, its causes, and its consequences. Features of the app supported participants in learning hand exercises and new working methods. Together with altered illness perception, this enabled participants to develop new behaviors. For a few participants, challenges such as pain during or after exercise were noted, indicating that some individuals may require more individualized face-to-face follow-up.

Patients with hand osteoarthritis represent a large patient group with limited access to recommended treatment. There is, therefore, a need for a new model for hand osteoarthritis care. The findings of this study demonstrate that participants benefited from the Happy Hands app. The app proved to be a valuable tool in empowering participants to better self-manage their condition. The result of this study suggests that the app can serve as a component in a treatment pathway for people with hand osteoarthritis.

## Data Availability

The dataset generated in this study is not available due to ensure participants anonymity and to not reveal sensitive information.

## Appendix

Multimedia Appendix 1 Interview guide.

Multimedia Appendix 2 COREQ checklist.

## Abbreviations

COREQ: Consolidated Criteria for Reporting Qualitative Research
RCT: randomized controlled trial
SCT: social cognitive theory
USIT: The University Information Technology Center
