# Supplementary material for: Changing Habits With the Happy Hands App: Qualitative Focus Group Study of a Hand Osteoarthritis Self-Management Intervention
Source: J Med Internet Res. 2026 Feb 2;28:e82773. doi: 10.2196/82773 (PMC12910265; doi:10.2196/82773)
Supplement: Multimedia Appendix 1 [file jmir_v28i1e82773_app1.docx]

# **Intervjuguide – intervju med deltagere som har brukt appen Happy Hands**

1. Først tenkte vi å ta en runde der alle kan presentere seg, og fortelle hvor lenge dere har hatt håndartrose.
2. Kan du si litt om hva du har fått av behandling for håndartrose før du begynte med appen?
3. Kan du si litt om hva du har fått av informasjon om håndartrose før du begynte med appen?
4. Nå har dere brukt appen i 3 måneder. Kan dere si litt om hvordan dere har opplevd å bruke appen?
5. Hvordan var det å sette i gang med å bruke appen?
   1. Fikk du råd/støtte?
6. Er det noe du tenker annerledes om eller gjør annerledes som følge av at dere har brukt denne appen?
   1. Har du noen eksempler fra hverdagen der du gjør ting annerledes etter at du begynte å bruke appen?
   2. Tenker dere annerledes om artrosen?
7. Har appen hjulpet dere på noen måte?
   1. Bedring/forverring?
8. Er det noe annet av innholdet i appen som har vært nyttig for dere? (dere har sagt en del om hvordan appen har vært nyttig for dere. Har dere noe mer å si om det? Har dere noen eksempler?)
9. Hvordan er det å bruke en app/teknologi som helsehjelp?
   1. Hvordan har det vært å bruke en app til å gjøre treningsøvelser?
10. Hva er dine tanker om å fortsatt bruke appen - hva er i så fall den viktigste grunnen til at du fortsatt vil eller vil ikke bruke den?
11. Er det noe du ikke liker eller skulle ønske var annerledes?
12. Er det noe som har vært utfordrende å forstå eller få til?
13. Er det noe du savner i appen?
14. Kan dere forklare hvordan dere har brukt appen?
    1. påminnelser
    2. treningsfilmer
    3. informasjonsfilmer
    4. quizzer
    5. grafer
    6. innstillinger
15. Har dere noen idé om hvordan vi kan gjøre denne appen kjent for personer med håndartrose og helsepersonell?
    1. Har du noen tanker om hvordan denne appen kan nå ut til enda flere?
16. (Hvis du skulle anbefale en du kjenner som har håndartrose å bruke app’en, hva ville du si da?)
17. har dere noen tanker om navnet på appen, Happy Hands?
18. har dere noen tanker om hva dere ville ha betalt om appen hadde kostet penger?
    1. Hvilken pris?

Nå har vi fått velig mye god informasjon fra dere og vi er snart ferdige.

1. Er det noe du vil føye til før vi avrunder?
